# Use of a Driving Simulator to Facilitate Older People to Return to Driving

**DOI:** 10.1093/geroni/igaa057.2595

**Published:** 2020-12-16

**Authors:** Carolyn Unsworth, Megan White, Natasha Lannin

**Affiliations:** 1 Central Queensland University, Melbourne, Victoria, Australia; 2 Alfred Health - Caulfield Hospital, Caulfield, Victoria, Australia; 3 Monash University, Melbourne, Victoria, Australia

## Abstract

Driving simulators are a relatively underutilized therapy tool that provide an opportunity for older drivers with a range of health-related problems to participate in simulated driving scenarios in a low cost and safe environment. The aim of this paper is to (i) describe the use of a Forum 8 driving simulator prior to a driver assessment, (ii) detail the story-boarding technique used to develop and grade driving scenes to enable older drivers to increase confidence, practice using vehicle modifications such as a spinner knob (e.g. for one-handed driving following stroke), and train specific skills including visual scanning and attention, and (iii) present five case studies to identify the strengths and limitations of incorporating the simulator into therapy programs with older drivers. of simulator use. The establishment and use of a driving simulator in a rehabilitation unit highlights both the challenges and benefits of using this kind of technology in practice. Part of a symposium sponsored by Transportation and Aging Interest Group.

